# An in vitro model for assessment of SARS-CoV-2 infectivity by defining the correlation between virus isolation and quantitative PCR value: isolation success of SARS-CoV-2 from oropharyngeal swabs correlates negatively with Cq value

**DOI:** 10.1186/s12985-021-01542-y

**Published:** 2021-04-07

**Authors:** Sissy Therese Sonnleitner, Julian Dorighi, Bianca Jansen, Carmen Schönegger, Sarah Gietl, Stephan Koblmüller, Christian Sturmbauer, Wilfried Posch, Gernot Walder

**Affiliations:** 1 Medical Laboratory, Department of Virology, Dr. Gernot Walder GmbH, 9931 Außervillgraten 30, Austria; 2 Institute of Biology, University of Graz, Universitaetsplatz 2, 8010 Graz, Austria; 3 Institute of Hygiene and Medical Microbiology, Medical University of Innsbruck, 6020 Innsbruck, Austria

**Keywords:** SARS-CoV-2, Infectivity, Transmission pattern in vitro, Quantitation of viral infectivity

## Abstract

**Background:**

At the beginning of the pandemic caused by severe acute respiratory syndrome coronavirus 2 (SARS-CoV-2), little was known about its actual rate of infectivity and any COVID-19 patient positive in laboratory testing was supposed to be highly infective and a public health risk factor.

**Methods:**

One hundred oropharyngeal samples were obtained during routine work flow of testing symptomatic persons by quantitative polymerase chain reaction (qPCR) and were inoculated onto cell culture of VeroB4 cells to study the degree of infectivity of SARS-CoV-2 in vitro. Quantification by virus titration and an external standard using synthetic RNA gave the breaking point of infectivity in SARS-CoV-2 in vitro.

**Results:**

A clear negative correlation (r = − 0.76; p < 0.05) could be asserted between the viral load in quantitative polymerase chain reaction (qPCR) and the probability of a successful isolation in serial isolation experiments of specific oropharyngeal samples positive in qPCR. Quantification by virus titration and an external standard using synthetic RNA indicate a Cq between 27 and 30 in E-gene screening PCR as a breaking point in vitro, where infectivity decreases significantly and isolations become less probable.

**Conclusions:**

This study showed that only the 21% of samples with the highest viral load were infectious enough to transmit the virus in vitro and determined that the dispersion rate in vitro is surprisingly close to those calculated in large retrospective epidemiological studies for SARS-CoV-2. This raises the question of whether this simple in vitro model is suitable to give first insights in dispersion characters of novel or neglected viral pathogens. The statement that SARS-CoV-2 needs at least 40,000 copies to reliably induce infection in vitro is an indication of its transmissibility in Public Health decisions. Applying quantitative PCR systems in diagnosis of SARS-CoV2 can distinguish between patients providing a high risk of transmission and those, where the risk of transmission is probably limited to close and long-lasting contacts.

## Introduction

SARS-CoV-2 was first described in December 2019 in Wuhan/China [17] and rapidly spread over many countries, including Thailand, Japan, Korea, Vietnam, Singapore [8, 10, 14, 16] and Italy [11], until it reached Austria not even three months later.

Austria was one of the countries with the lowest infection rate in the first outbreak of SARS-CoV-2, which started on 25th of February with the first two documented cases and hit its peak on 26th of March 2020, ten days after the official lockdown. That Austria got off relatively lightly, can be attributed to the early lockdown on 16th of March and the strict policies involved. Governmental relaxations have been progressing since May 2020, with Austria slowly opening up again.

Viral transmission dynamics are often described statistically using the reproduction number R_0,_ a summary measure of the transmissibility of infectious diseases [4], reflecting the average number of infected persons per carrier. Indeed, SARS-CoV-2—like MERS and SARS—seems to create inhomogeneous transmission dynamics, where a certain amount or a majority of transmissions can be traced back to a minority of carriers, reflected by the overdispersion parameter *k*. *k* is calculated especially high in MERS (0.06, 95% CI) and rather high in SARS1 (0.20; 95% CI), which means that the majority of infections might be traced back to 6% and 20%, respectively, of the infected persons [3]. Calculations of *k* in SARS-CoV-2 vary between 0.1; 95% CI [6], 0.45, 95% CI [1] and 0.67, 95% CI [19]. However, these authors point to some uncertainty in data and calculations [6].

Several recent studies take the meaning of Super-spreading events (SSE) into account for a score or even the majority of SARS-CoV-2 transmissions [1, 6, 18, 19]. The use of the dispersion parameter *k* contributes much better to the understanding of transmission than the reproduction number R_0_.

In this study, we tried to isolate SARS-CoV-2 by culturing every PCR-positive swab during the first acute outbreak situation in our region and found that only the 21% of samples with the highest viral load were infectious enough to transmit the virus in vitro and determined that the dispersion rate in vitro is surprisingly close to those calculated in large retrospective epidemiological studies for SARS-CoV-2. This raises the question of whether this simple in vitro model is suitable to give first insights in dispersion characters of novel or neglected viral pathogens and of whether the dispersion parameter *k* describes the dispersion pattern of SARS-CoV-2 more realistic and contributes much better to the understanding of transmission than the reproduction number R_0._ Furthermore, we quantified the infectious dose of SARS-CoV-2 in vitro by virus titration and an external standard using synthetic RNA.

## Material and methods

### Sample collection

109 oropharyngeal samples were obtained during routine workflow of testing symptomatic persons or those exposed to them [7]. Viral RNA was extracted using automated IndiMag 48 and an IndiMag Pathogen kit in accordance with the manufacturer's instructions (Indical Bioscience GmbH, Germany).

### qPCR

Extracts were tested for SARS-CoV-2 by qRT-PCR using the Bio-Rad CFX96 system (Roche, Switzerland) with a LightMix Modular Assay kit in accordance with the modified Charité guidelines [5]. 10 µl of extracted RNA were added into 15 µl reaction mixture (mastermix). Each 15 µl mastermix contained 12.5 µl buffer solution, 0.25 µl enzyme mix, 1.75 µl of nuclease-free water and 0.5 µl primer probe wHCoV (E-Gene, as well as N-Gene and Rdrp-Gene for confirmation). Reactions were incubated at 55 °C for 5 min and 95 °C for 5 min in order to conduct reverse transcription of viral RNA, sample denaturation and enzyme activation. These steps were followed by PCR-amplification including 45 cycles at 95 °C for 5 s, 60 °C for 15 s and 72 °C for 15 s. Cooling was implemented at 40 °C for 30 s.

Results were interpreted based on the Second derivative maximum (SDM) method. Positive results were confirmed by Rdrp and N-gene [5], samples with an initial Cq value lower than or equal to 37 were assigned to repeated testing including extraction. A Cq value higher than 40 was defined negative. All positive samples appearing for the first time were collected from 3rd of April until 16th of May and stored in the refrigerator for further isolation.

### Isolation of SARS-CoV-2

In the course of routine diagnosis, every sample positive for SARS-CoV-2 in qPCR was taken for isolation in cell culture over a period of seven weeks in April and May 2020. Isolation of SARS-CoV-2 was performed from oropharyngeal and stool samples after positive specific qPCR by inoculation on VeroB4 (no. ACC-33, DSMZ) in T25 tissue culture flasks (Sarstedt, Germany) for 1 h at 36 °C. After incubation, the sample was removed and Medium199 (Gibco, USA) with 2.5% fetal calf serum (FCS; Gibco, USA) and a mixture of antibiotics was added (streptomycin, vancomycin, penicillin, each 1 µg/ml). We monitored virus cultures daily for cytopathic effects and tested for specific viral RNA every three days. Isolation was considered successful when cytopathic effect was 80 to 100% in passage 0 as well as passage 1 and/or Cq value in qPCR was lower than 15. Highly positive supernatants were harvested, centrifugated at 13.000 rpm for 5 min and stored at − 80 °C in 10% FCS. A further passage of diverse isolates was performed to obtain the highest possible concentration, which lay at Cq 14 on average. All work involving infectious SARS-CoV-2 was carried out in a BSL3 facility, following the institutional guidelines and regulations.

### The viral infectivity in samples stored at different temperatures:

To determine whether long storage effects the initial virus last in our samples, two isolates of SARS-CoV-2 were deposited in Medium199 in 1.5 ml tubes (Eppendorf, Germany) in the refrigerator (4 °C), incubator (36 °C) and at room temperature (20 °C, in the BSL-3 safety cabinet) for 15 days and tested for the presence of viral specific RNA by qPCR weekly.

### Virus titration

Confluent VeroB4 cells were cultured in Medium199 including 5% FCS in T75 tissue culture flasks (Sarstedt, Germany) and transferred into 96-well tissue culture plates (Sarstedt, Germany). Passage 1 isolates of SARS-CoV-2 were thawed from − 80 °C freezer and titrated from 1:10 to 1:10^−12^ in U-shaped 96-well plates (Greiner, Germany) and pipetted into each corresponding well of the 96-well tissue culture plate. Plates were incubated at 36 °C. Three days post infection, incubation was stopped by gently removing the supernatant, washing the cells three times with PBS and fixing cells in 1:1 ice-cold acetone-methanol. For easier optical evaluation, cells were dyed by crystal violet staining and tissue culture infectious dose of 70% (TCID_70_) and plaque-forming units (PFU) were calculated [15]. Titration was performed twice with our isolates no. I1 and I2.

## Results

**Fig. 1:**
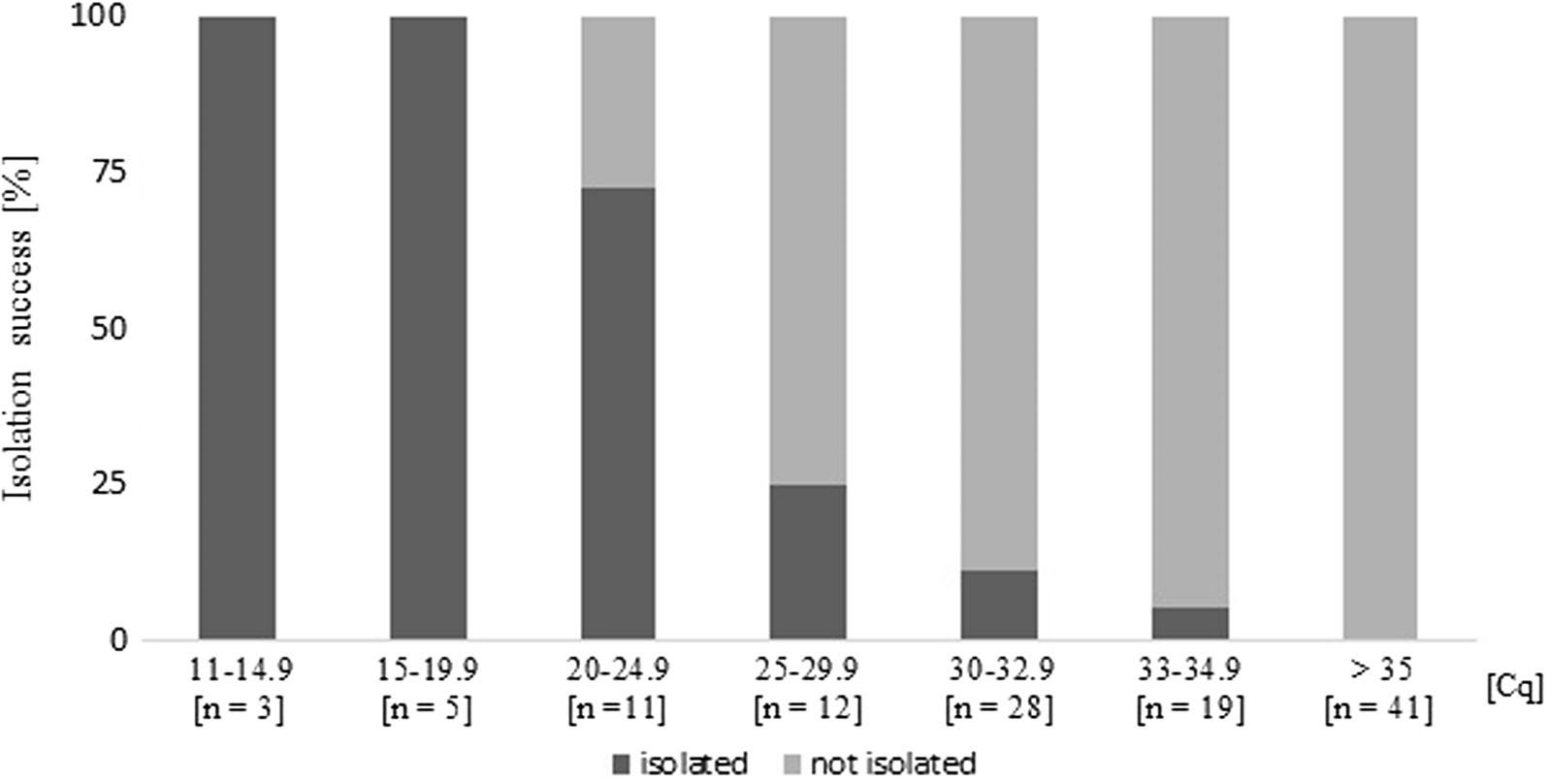
Success of isolation of SARS-CoV-2 in VeroB4 cells by Cq-values (%). Isolation success correlated negatively with the initial Cq value in the sample (r = − 0.76; p < 0.05). All oropharyngeal samples with an initial viral load lower than Cq 20 in E-gene screening PCR (n = 8), could be grown in vitro (100%). An initial Cq value between 20 and 24.9 led to a 72.7% chance of infection, which decreased to 25% at an initial viral load between 25 and 29.9 and to 7.1% at an initial load between 30 and 34.9. Samples with initial viral loads of Cq 35 or higher could not be isolated in our experiment

109 SARS-CoV-2 positive samples were inoculated on VeroB4 cells in April and May 2020, due to fungal contaminations, 9 flasks had to be discarded before evaluation. Of the remaining 100 trials, 21 (21%) were successful, as shown in Fig. 1. On average, isolation could be detected by cytopathic effect and subsequent qPCR 4.4 days post infection. The lowest Cq values, 14.6 and 13.33, respectively, were detected in the samples from two patients in May 2020. Both samples were isolated within three days. The shortest period until isolation was three days in four cases (19%), the longest period was 13 days, concerning the oropharyngeal sample of a recurrent patient nearly 2 weeks post infection and after a blind passage. The same sample (R2) was re-thawed after the first successful isolation and could be re-isolated, but much faster after four days in the second trial. This second isolation was not taken into account in our isolation experiments. No isolate could be obtained in cell culture with stool sample, even though the initial Cq value was rather low (23.13).

Isolation success correlated negatively with the initial Cq value in the sample (r = − 0.76; p < 0.05). All oropharyngeal samples with an initial viral load lower than Cq 20 in E-gene screening PCR (n = 8), could be grown in vitro (100%). An initial Cq value between 20 and 24.9 led to a 72.7% chance of infection, which decreased to 25% at an initial viral load between 25 and 29.9 and to 7.1% at an initial load between 30 and 34.9. Out of 41 samples with Cq 35 or higher, we could not isolate any (Fig. 1).

The only two samples that could be isolated despite higher Cq values were R1 (Cq 32.15) and R2 (Cq 30.48), the last sample twice. R2 was the swab of a recurrent patient 20 days after the first positive swab and needed at least 13 days and a blind passage until successful isolation. To confirm this result, we thawed the original sample R2 from − 80 °C and tried to isolate again, which, surprisingly, was successful within a week. The results were confirmed by different SARS-CoV-2 specific PCRs as well as an Immunofluorescence assay (IFA). Whole genome sequencing for identifying the virus strain is under way to clarify this case. Separate from those two special samples R2 and R1, isolation was successful in VeroB4 cell culture within a week and with 81.1% of the samples with viral loads lower than Cq 27.0 in E-gene qPCR. (18 of 22).

### Overall distribution of Cq values during the outbreak

**Fig. 2:**
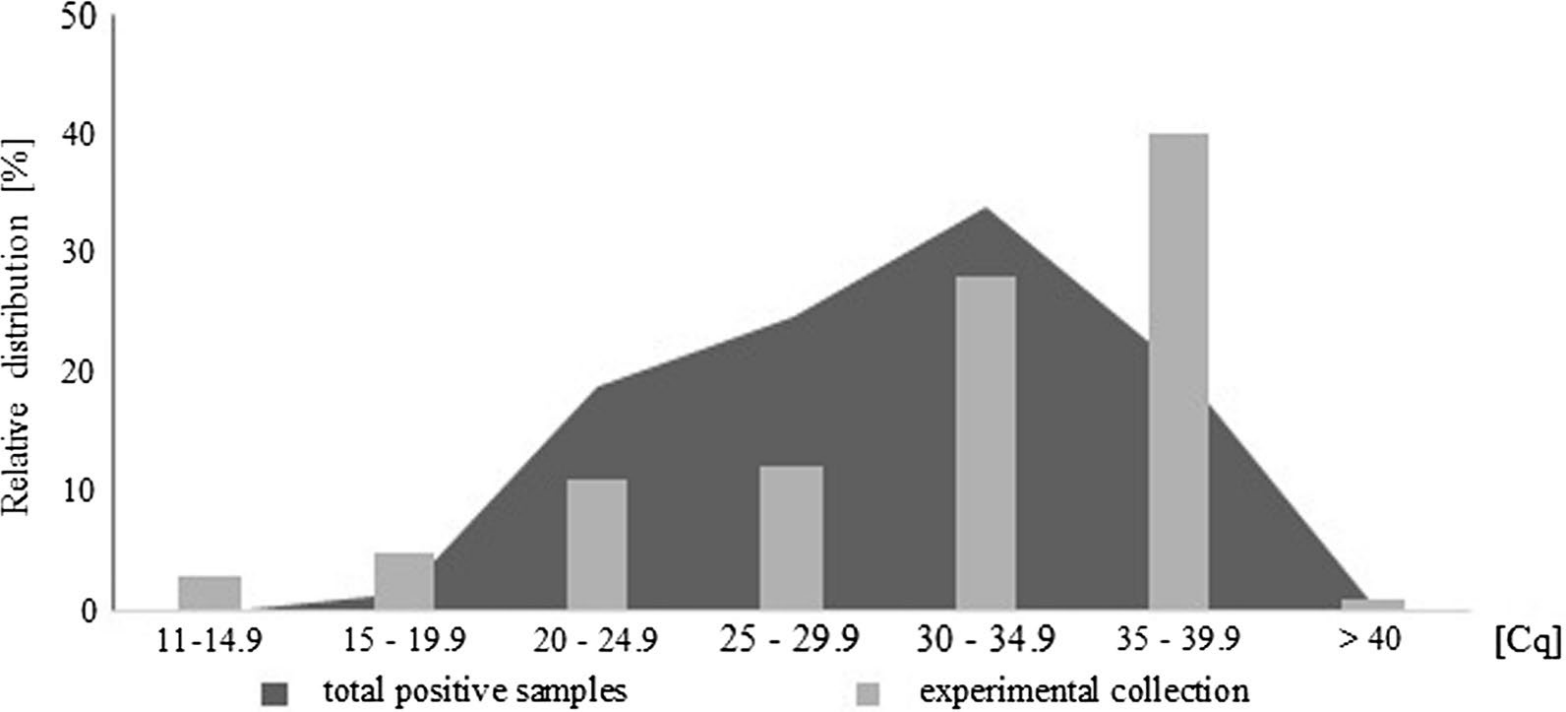
The distribution of Cq values during the outbreak in a total data set of 371 samples (dark grey). The light grey bars show the comparable distribution of Cq values in our experimental sample collection and demonstrate that our sample set is representative for the distribution of initial viral loads during an outbreak of SARS-CoV-2

Besides the higher number of samples with a Cq of 35–39.9, the relative distribution of viral load is comparable between our sample (n = 100) and the whole amount of SARS-CoV-2 positive samples acquired during the first outbreak n = 371), as illustrated in Fig. 2.

Infection of cell cultures with specific positive samples was carried out twice a week. To ensure no loss of viral load during storage, we monitored two stored samples as well as two negative controls (distilled water and a cell culture supernatant; these tend to give wrong positive signals in E gene qPCR due to unspecific reactions with primate RNA, which was tested by sequencing) for two weeks by qPCR.

Storage of samples at 18 °C respectively 36 °C led to a loss of viral load from original Cq 13 (1.1 × 10^5^ TCID_70_) to Cq 15 (5 × 10^4^ TCID_70_) and Cq 24.4 (120 TCID_70_), which means a loss of 54.4% respectively 89.1%. No significant loss of viral RNA could be detected in samples stored at 4°, where the viral load stayed at Cq 13 (1.1 × 10^5^ TCID_70_).

Effective viral load was determined by quantification via virus titration. Confluent monolayers of VeroB4, seeded in 96-well plates, showed cytopathic effects of 70% or more three days post infection with viral concentrations from 10^−1^ to 10^−4^ as well as in 6 of 16 wells of 10^−5^. No cytopathic effect was observed in wells infected with viral dilutions of 10^−6^ or lower. Calculating the tissue culture infectious dose of 70% (TCID_70_) [15] resulted in 1.1 × 10^6^ TCID_70_, which corresponds to 1.1 × 10^6^ TCID_70_.

**Fig. 3:**
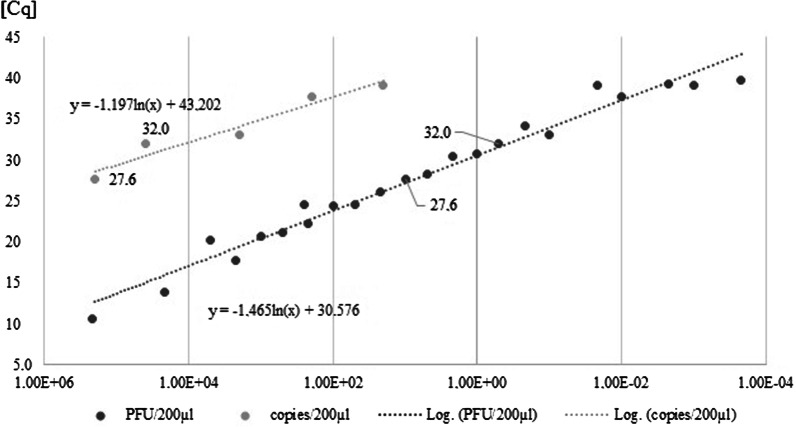
Dilution series of plaque-forming units (PFU) of an average of our isolates compared to a commercially available external standard using synthetic RNA copies (COV019, CE, Exact Diagnostics, USA)

Additionally, virus titration showed that infection of cell culture was still possible in 37.5% (6 of 16) at a dilution of 10^−5^, corresponding to 11 plaque-forming unites (PFU; 11 TCID_70_) per ml, but not at a dilution of 10^−6^ (1.1 PFU per ml). As shown in Fig. 3, those data correspond exactly to our data obtained from the in vitro model: Cq 26.16 shows 110 TCID_70_, a viral load, where isolation and infection are rather predictable (81.8%). Cq 30.48 reflects a viral load of about 11 TCID_70_, a viral concentration where isolation was only achieved with higher effort and occasionally (2.9% with considerable effort).

## Discussion

The fact that higher initial viral load leads to a higher isolation success is not surprising and well-known. However, the significance and predictability shown in our data set of at least 100 samples is astonishing and fits perfectly to actual data concerning transmission patterns of SARS-CoV-2 [1, 6]. We assume a Cq value between 27 and 30 in E-gene screening PCR as a breaking point (11 to 110 TCID_70_) for in vitro infection, where infectivity decreases significantly (p < 0.05) and transmission becomes less probable.

Our data revealed that 21% of our samples were infectious enough to transmit the virus in vitro, which perfectly fits to a recently published cluster analysis from Hong Kong, where Super-spreading events (SSE) were analysed and 20% of the infections led to the main component of transmissions [1]. Therefore, the use of the dispersion parameter *k* contributes much better to the understanding of transmission than the reproduction number R_0_ and Public health policies can be adapted by discovering and preventing typical situations, where SSEs occur. An SSE or overdispersion emerges in conjunction with immune-suppressive patients, increased disease severity and therefore viral load, asymptomatic individuals and extensive social interactions [2]. Additionally, future transmissions will depend on factors including the degree of seasonal variations in transmission, the duration of immunity, and the degree of cross-immunity between SARS-CoV-2 and other coronaviruses as well as the intensity and timing of control measures [9].

Isolation was also possible in the case of a recurrent patient, even though 13 days of incubation on cell culture were needed to obtain cytopathic effect and SARS-CoV-2 specific positive PCR for confirmation of successful isolation.

Besides the higher number of samples with a Cq of 35–39.9, the relative distribution of viral load is comparable between our sample (n = 100) and the whole amount of SARS-CoV-2 positive samples acquired during the first outbreak (n = 371). Therefore, in question of the initial viral load, our data reflect the distribution of an outbreak situation realistic.

Our quantitative comparison between an external standard using synthetic RNA and plaque-forming units received via virus titration showed the imperative necessity to distinguish between the both of it and carefully choose the type of quantitative standard. For example, Cq 32,56 shows 1 PFU/200 µl (1 TCID_70_) and simultaneously the difference of 40,000 copies/ml, suggesting an enormous number of RNA copies in a cell culture supernatant and probably also in an oropharyngeal sample, which is not viable, at least not possible to perform cytopathic effects and perhaps represent just fragmentary and ineffective RNA-copies.

We are aware of the limitations of our in vitro model, especially the circumstance, that we cannot suppose the same level of susceptibility between our cell line and the environment of the human oropharyngeal zone. Another limitation is caused by the use of only one cell line VeroB4, a line which is sufficiently susceptible to SARS-CoV-2, but seems to be inferior to the cell line VeroE6 due to the lower content of the cell surface protein TMPRSS2 (Transmembrane serine protease 2) [13]. Further studies concerning serial isolation experiments on different cell lines simultaneously would be desired to determine the influence of the cell line on the experimental approach.

However, it is a fair approximation of viability. Our data reveal that 21% of our samples were infectious enough to transmit the virus in vitro, with the probability of a positive culture decreases steeply above a Cq value of 27, but transmission may still occur in patients with very close and long-lasting contact, especially household transmissions [12] and of course it must not be overseen that the viral load may vary over the duration of the infection and that the Cq value only represents a short-term current status of the infectivity.

Storage in the refrigerator for 1 week does not affect the initial viral load significantly, which confirms the high stability of an enveloped virus and facilitates the interpretations of patient’s material stored for some days before further handling. Actually, a significant loss of viral titer due to storage in the incubator under the same conditions apart from the temperature, was an interesting result. Outbreak situations are characterized by shortage of material and restrictions affecting transport and storage, so it is important to know that prolonged storage at room temperature has no major effect on PCR testing.

With regard to the distancing and hygiene rules, the viral shedding and therefore, the risk of transmission, should be minimal at a viral concentration of 11 TCID_70_ and lower.

If the assumption is correct and the meaning of SSEs in transmission of SARS-CoV-2 is as high as supposed, it will be the greatest challenge in the near future to find out which policies effectively tackle the dispersion, in ideal circumstances without the hard restrictions of a total lockdown.

In this respect, our data help to take well-directed precautions and Public health policies to prevent SSEs and simultaneously, relax measures that seem to have rather a psychological than an epidemiological effect. Applying quantitative PCR systems in diagnosis of SARS-CoV-2 can distinguish between patients providing a high risk of transmission and those, where the risk of transmission is probably limited to close and long-lasting contacts. Unlike medically indicated sick leave, quarantine represents a measure restricting liberty that must be well considered and should be imposed in the most moderate way. In further outbreaks, authorities will need a reliable diagnostic reason to impose restrictions that fit both the constitutional right of personal freedom and the needs of public health.

## Conclusion

This study could show that the amount of 100 TCID_70_ (Cq 27 in E gene screening PCR) of viral load will guarantee a successful isolation of SARS-CoV-2 in VeroB4 cell culture.

Infection in vitro mandatorily needs a specific viral load, which gives high insights into infectivity of SARS-CoV-2 and additionally gives reference points for diagnostic laboratories and medicines, where patients are highly infectious and have to stay in home quarantine and where relieves in strict home quarantine are conceivable. The statement that SARS-CoV-2 needs at least 40,000 copies to reliably induce infection in vitro might serve as an indication of its transmissibility in Public Health decisions. Applying quantitative PCR systems in diagnosis of SARS-CoV2 can distinguish between patients providing a high risk of transmission and those, where the risk of transmission is probably limited to close and long-lasting contacts.

## Data Availability

All data generated or analysed during this study are included in this published article.

## Abbreviations

COVID-19: Coronavirus induces disease 2019
FCS: Fetal calf serum
*k*: Overdispersion parameter k
PFU: Plaque forming units
qPCR: Quantitative polymerase chain reaction
qRT-PCR: Quantitative reverse transcriptase polymerase chain reaction
R: Reproduction factor
RNA: Ribonucleic acid
SARS1: Severe acute respiratory syndrome coronavirus 1
SARS-CoV-2: Severe acute respiratory syndrome coronavirus 2
SDM: Second derivative maximum
SSE: Super-spreading events
TCID: Tissue culture infectious dose
TMPRSS2: Transmembrane serine protease 2
