# Correction to: An in vitro model for assessment of SARS‑CoV‑2 infectivity by defining the correlation between virus isolation and quantitative PCR value: isolation success of SARS‑CoV‑2 from oropharyngeal swabs correlates negatively with Cq value

**DOI:** 10.1186/s12985-021-01558-4

**Published:** 2021-04-20

**Authors:** Sissy Therese Sonnleitner, Julian Dorighi, Bianca Jansen, Carmen Schönegger, Sarah Gietl, Stephan Koblmüller, Christian Sturmbauer, Wilfried Posch, Gernot Walder

**Affiliations:** 1 Medical Laboratory, Department of Virology, Dr. Gernot Walder GmbH, 9931 Außervillgraten 30, Austria; 2 Institute of Biology, University of Graz, Universitaetsplatz 2, 8010 Graz, Austria; 3 Institute of Hygiene and Medical Microbiology, Medical University of Innsbruck, 6020 Innsbruck, Austria

## Correction to: Virol J (2021) 18:71 https://doi.org/10.1186/s12985-021-0142-y

Following publication of the original article [1], we have been informed that all the authors have the first names and surnames interchanged.

The author group has been updated above and the original article has been corrected.
